# Validity and reliability of the Arabic version of the patient's joint perception question in patients undergoing knee arthroplasty

**DOI:** 10.1002/jeo2.70402

**Published:** 2025-08-31

**Authors:** Khalid A. Alsheikh, Firas M. Alsebayel, Abdulrahman A. Alzahrani, Bader K. Alqahtani, Jude N. Abanmi, Abdulaziz F. Altammami

**Affiliations:** ^1^ College of Medicine King Saud bin Abdulaziz University for Health Sciences Riyadh Saudi Arabia; ^2^ Department of Orthopedic Surgery National Guard Health Affairs Riyadh Saudi Arabia; ^3^ Research Department King Abdullah International Medical Research Center Riyadh Saudi Arabia

**Keywords:** arthroplasty, joint perception, patient‐reported outcome, reliability assessment, translation and validity

## Abstract

**Purpose:**

Total knee arthroplasty (TKA) aims to alleviate pain and restore function in patients with knee osteoarthritis. While the Forgotten Joint Score (FJS) and Western Ontario and McMaster Universities Osteoarthritis Index (WOMAC) are established measures of patient satisfaction and functional outcomes after TKA, they may not directly capture the patient's subjective perception of the joint itself. The FJS was used to assess concurrent validity, as it reflects the degree to which patients are unaware of their artificial joint, which is related to joint perception. The patient's joint perception (PJP) question offers a simplified alternative to evaluate joint awareness.

**Methods:**

This prospective observational study included patients who underwent TKA between 2018 and 2023. An Arabic version of the PJP (Ar‐PJP) question was translated using a forward‐backwards translation process. Participants completed the PJP, FJS, and reduced WOMAC at two time points, three weeks apart. Statistical analyses assessed validity and reliability using Pearson's correlation coefficient.

**Results:**

A total of 100 participants were included in the study. The mean PJP score was 28.9 (standard deviation [SD]: 13.7), and the mean WOMAC score was 46.1 (SD: 17.8). A moderate negative correlation was found between the Ar‐PJP score and FJS (*r* = −0.683; *p* < 0.001), A moderate negative correlation was found between the Ar‐PJP score and FJS (*r* = −0.683; *p* < 0.001), while the correlation with WOMAC was weak and non‐significant (*r* = −0.088; *p* = 0.382), supporting discriminant validity.

**Conclusions:**

The Ar‐PJP is a valid and reliable tool for assessing patients' perceptions post‐TKA. As a single‐question measure, it simplifies evaluations and enhances patient care in Arabic‐speaking populations.

**Level of Evidence:**

Level II.

**Abbreviations**
Ar‐PJP: Arabic version of patient's joint perception
CI: confidence interval
FJS: Forgotten Joint Score
PJP: patient's joint perception
PROM: patient‐reported outcome measure
r‐WOMAC: Reduced Western Ontario and McMaster Universities Osteoarthritis Index
SD: standard deviation
SPSS: Statistical Package for Social Studies
TKA: total knee arthroplasty
WOMAC: Western Ontario and McMaster Universities Osteoarthritis Index

## INTRODUCTION

Total knee arthroplasty (TKA) is a prevalent surgical intervention performed worldwide. The incidence of this procedure is anticipated to rise significantly over the next decade [11]. There are various methods for evaluating the outcomes of TKA. Traditionally, the surgeon serves as the primary assessor, utilizing criteria such as implant survival, complication rates and objective metrics including range of motion and walking distance [8]. As a result, measures known as patient‐reported outcome measures (PROMs), including the Western Ontario and McMaster Universities Osteoarthritis Index (WOMAC), which is a patient‐reported satisfaction measure, were created to evaluate patients' functionality and their level of satisfaction [3].

Recently, the Forgotten Joint Score (FJS) has been introduced [2] to improve the ceiling effect of other PROMS. This tool evaluates patients' awareness of their reconstructed joint by quantifying their ability to forget its presence. The perception of a forgotten joint signifies a harmonious integration of the prosthesis with the individual's body, characterized by a sense of ‘forgotten joint’. This state may represent the ultimate objective for patients undergoing joint reconstruction [6].

The evolving concept of joint perception has given rise to the patient's joint perception (PJP) question, a tool specifically designed to evaluate patients' perceptions of their prosthetic joints [5, 8]. By utilizing a single‐item PROM, the PJP question assesses how individuals perceive and appraise the functionality, comfort and overall integration of their prosthetic joint into their daily lives. This measure not only facilitates a deeper understanding of patient satisfaction and quality of life post‐surgery but also provides valuable insights into the psychological and functional aspects of joint replacement, ultimately guiding improvements in prosthetic design and patient care strategies [12]. While the PJP can be used independently, it can also complement other PROMs to provide a more comprehensive understanding of the patient's experience [6].

There has been no validated Arabic version of PJP (Ar‐PJP) question till now, and only a few studies have investigated the validity and reliability of the PJP question. This research aims to create a translated and cross‐culturally adapted Ar‐PJP question and test its cross‐cultural adaptation, validity and reliability.

## METHODS

This study is a prospective observational analysis of patients who underwent TKA from 2018 to 2023 due to degenerative knee osteoarthritis. This study was conducted at King Abdulaziz Medical City, Riyadh, Saudi Arabia. Patients were excluded from the review if they had undergone revision surgeries, had concomitant lower extremity disorders, or declined to provide consent. Ethical approval for the study (no. NRC24R/114/02) was granted by the Institutional Review Board (IRB) of King Abdullah International Medical Research Center (KAIMRC). Consent was obtained from each participant during follow‐up clinic visits, where they were informed about the study's objectives and their right to withdraw at any time without repercussions. To protect participant confidentiality, each participant was assigned a unique study ID that was used to match their test and retest responses. No personally identifiable information was collected. Participants received no monetary incentives or rewards for their involvement. A single research member administered the test‐retest survey via telephone, reading the question verbatim to each participant to ensure consistent delivery and minimize variability. Participants were given ample time to consider their response before answering. Once the survey process began, researchers only intervened to clarify questions if explicitly requested by the participant. The average duration of each telephone survey was approximately 15 min. Data for the test and retest were collected between August 2024 and September 2024, with a 3‐week interval between assessments. The participants were all of Saudi Arabian descent and native Arabic speakers with the same Arabian dialect.

### Patient characteristics and inclusion and exclusion criteria

The study population consisted of adult patients undergoing primary TKA. The mean age of the participants was 56 ± 3 years. All participants were of Saudi Arabian descent and native Arabic speakers. A total of 59 female and 41 male participants were included in the study. Patients were included if they: (1) were scheduled for primary TKA due to osteoarthritis; (2) were able to understand and complete the questionnaires in Arabic and (3) provided informed consent to participate in the study. Patients were excluded if they: (1) had undergone previous revision TKA; (2) had concomitant lower extremity disorders that could affect joint perception; (3) had cognitive impairment that would preclude accurate questionnaire completion or (4) declined to provide consent.

### Patient‐reported outcome tools

The PJP question is a single‐item questionnaire developed by Puliero et al. to assess a patient's subjective perception of their artificial joint [8]. It aims to measure the degree to which the patient perceives their joint as feeling natural and integrated into their body. The PJP uses a single question with five possible answers, rating from the highest expected result, ‘like a native or natural joint’, to the worst possible case, ‘a non‐functional joint’.

The FJS consists of 12 questions designed to evaluate joint awareness in daily life and is widely used in research on lower limb arthroplasty. This study utilized a validated French‐Canadian version of the FJS, where scores range from 0 (worst outcome) to 100 (best outcome).

The WOMAC includes 24 questions categorized into three domains: pain (5 questions), stiffness (2 questions) and function (17 questions). It is a widely recognized tool for assessing outcomes in patients with lower limb osteoarthritis or joint replacement. In this study, WOMAC scores were reported with 0 representing the best outcome and 100 the worst.

### Translation process

The PJP question was translated into Arabic utilizing the forward‐backwards translation method, carried out by native Arabic speakers with medical expertise. Subsequently, the research team conducted a pilot study involving 30 randomly selected patients who had undergone TKA to evaluate the clarity and comprehension of the questionnaire. After reaching a consensus among all authors, the final Arabic version was finalized, with no modifications made to the version provided by the original author.

### Validation process and data acquisition

In this study, participants completed the Arabic version of the PJP (Ar‐PJP) question at two distinct time points, separated by a 3‐week interval. Initially, each participant filled out the Ar‐PJP independently to provide an assessment of their joint awareness regarding their prosthetic knee. After the three‐week period, participants were asked to complete the Ar‐PJP once again, alongside the reduced WOMAC (rWOMAC) and the FJS [1]. This dual‐assessment approach aimed to evaluate the validity and reliability of the translated questionnaire by comparing the results across different measures of joint function and patient‐reported outcomes.

The PJP question: The PJP is a single‐item questionnaire where patients rate their perception of their joint on a scale of 1– 5 (1: Normal joint, 5: Prosthetic joint).

The FJS: The FJS is a 12‐item questionnaire assessing the ability to forget about the artificial joint during daily activities. Each item is scored on a 5‐point Likert scale, resulting in a total score ranging from 0 to 100, with higher scores indicating better joint function and less awareness of the joint.

WOMAC: The WOMAC is a widely used questionnaire assessing pain, stiffness and physical function in patients with osteoarthritis. It consists of 24 items, with 5 items for pain, 2 for stiffness and 17 for physical function.

### Statical analysis

The Statistical Package for Social Studies (SPSS 28; IBM Corp.) was used to analyse the data for this study. Pearson's correlation coefficient was used to assess the construct validity of the translated tool. Besides, Pearson's correlation coefficient was used to assess the strength and direction of the linear relationship between items of the questionnaires. This would provide a 95% confidence interval (CI). The level of statistical significance was considered to be a *p* value of <0.05.

## RESULTS

### Reliability of the Ar‐PJP

A total of 100 participants were involved in this study. The mean Ar‐PJP score was 28.9 (standard deviation [SD]: 13.7), and the mean WOMAC score was 46.1 (17.8). The mean Ar‐PJP score at the first time point and the second time point was 2.41 (1.2) and 2.45 (1.0), respectively. The mean FJS percentage was 66.4% (SD: 31.4%).

### Validity of the Ar‐PJP against WOMAC and FJS

**Figure 1:**
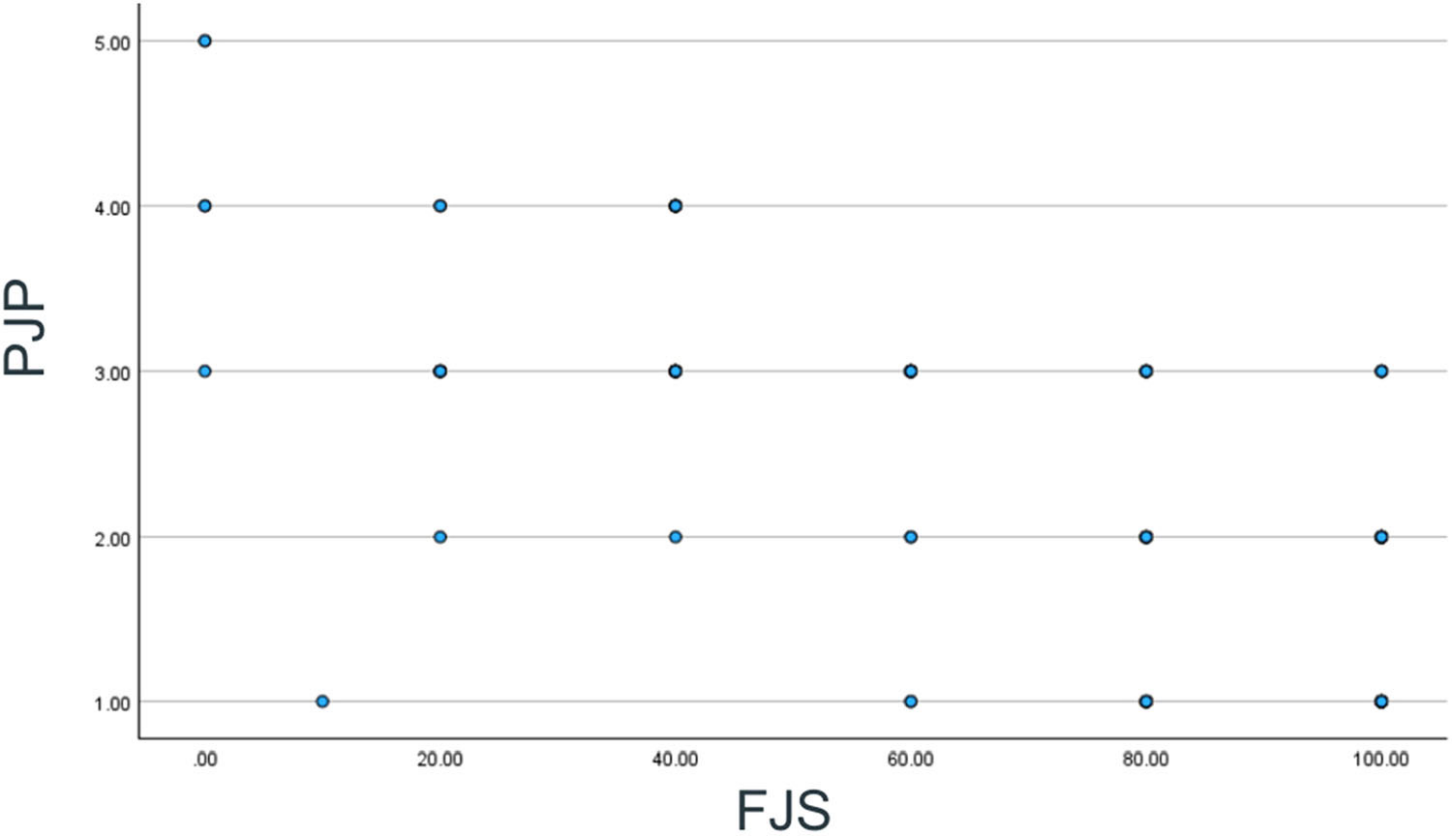
Correlation between PJP score and the FJS percentage. FJS, Forgotten Joint Score; PJP, patient's joint perception.

The correlation analysis between the Ar‐PJP at a 3‐week interval and the score for the WOMAC scale shows a weak negative correlation of −0.088 (95% CI: −0.279 to 0.109). However, this was not statistically significant (*p* value = 0.382). The correlation analysis between the Ar‐PJP score and the FJS shows moderate correlation of −0.683 (95% CI: −0.775 to −0.564) (Figure 1). This correlation between FJS and PJP score would indicate that these measures are closely related and are useful in the assessment of outcomes in patients with knee osteoarthritis or post‐arthroplasty in the Arabic‐speaking population.

### Reliability analysis

– Internal consistency was not assessed as the PJP is a single‐item measure.
– Test–retest reliability was assessed using Cohen's weighted kappa (*κ*) to determine the agreement between PJP scores at the two time points.

### Validity analysis

– Content validity was established during the translation and cross‐cultural adaptation process, as described in Figure 1.
– Discriminant validity was assessed by examining the correlation between the PJP and WOMAC using Pearson's correlation coefficient.
– Criterion validity was assessed by examining the correlation between the PJP and FJS using Pearson's correlation coefficient.

## DISCUSSION

The current study successfully translated and validated the Ar‐PJP question for patients undergoing TKA. This work is significant because it provides Arabic‐speaking patients with a validated tool for assessing their perceptions of joint functionality and overall satisfaction post‐surgery, an area that has previously been underserved. Given the rising prevalence of knee arthroplasties in Arabic‐speaking populations, culturally sensitive assessment tools are crucial for enhancing patient care and outcomes.

A central aim of this study was to introduce the PJP as a streamlined, one‐question tool to replace multiple traditional PROM assessment tools, such as the FJS and WOMAC. While these conventional PROMs have been instrumental in evaluating surgical outcomes, they often involve multiple questions that can contribute to patient burden and may possess significant ceiling effects. These limitations hinder their ability to fully capture the holistic patient experience post‐TKA [5, 8].

Our findings revealed that the Ar‐PJP effectively correlates with established PROMs like FJS, demonstrating that a single‐item question can offer insights that are comparable to multi‐item PROMs. Specifically, we observed a correlation of *r* = −0.683 between the Ar‐PJP and FJS, consistent with findings by Behrend et al., who indicated that the FJS is crucial for measuring joint awareness and patient satisfaction following arthroplasty [2]. Additionally, the lack of correlation with WOMAC suggests that the PJP measures a construct distinct from the pain, stiffness and functional limitations assessed by the WOMAC, supporting the discriminant validity of the PJP [1].

Such findings highlight the potential for the PJP to address the shortcomings of traditional PROMs. For instance, studies by Varacallo et al. and Puliero et al. demonstrated that patients with optimal WOMAC score and FJS may still report feelings of joint awareness, reflecting the complexity of joint perception that these conventional tools fail to capture fully [8, 10]. In contrast, the PJP narrows in on the essential aspect of joint integration, focusing directly on a patient's subjective experience of their joint's presence in daily life, which is more relevant to overall satisfaction and quality of life post‐TKA [5, 7].

Moreover, the integration of the PJP into clinical practice could help reduce the patient burden associated with lengthy questionnaires, which have been shown to decrease response rates and increase the risk of incomplete surveys [4, 9]. The simplicity of the PJP ensures that valuable information about joint perception is obtained without overwhelming the patient, thereby enhancing the quality of health research in joint arthroplasty.

This study has limitations. First, it is a single‐centre study with a limited sample size, which may affect the generalizability of the findings to other populations or healthcare settings. While the study population consisted of patients undergoing TKA at a single centre, the consistent results across different analyses suggest that the Arabic PJP is a promising tool. Second, while the FJS was used to assess concurrent validity, the availability of other validated measures of joint perception in Arabic is limited. Future studies could explore the use of alternative questionnaires to further validate the PJP. Moreover, further research is necessary to apply the scale in multiple languages and populations to confirm its broad applicability.

## CONCLUSIONS

In conclusion, this study demonstrates that the Arabic version of the PJP is a reliable and valid tool for assessing joint perception in Arabic‐speaking patients undergoing TKA. The PJP offers a brief and easily administered measure of a patient's subjective experience of their artificial joint. Further research is needed to explore the clinical utility of the PJP in various settings.

## AUTHOR CONTRIBUTIONS

All authors contributed significantly to this research. Conceptualization and study design were carried out collaboratively by Dr. Khalid A. Alsheikh, Dr. Firas M. Alsebayel and Dr. Abdulrahman A. Alzahrani. Data collection was performed by Dr. Bader K. Alqahtani and Dr. Jude N. Abanmi. Data analysis and interpretation were conducted by Dr. Abdulaziz F. Altammami and Dr. Firas M. Alsebayel. The manuscript was drafted by Dr. Khalid A. Alsheikh and Dr. Abdulaziz F. Altammami, with all authors reviewing and approving the final version. All authors have agreed to be accountable for all aspects of the work.

## CONFLICT OF INTEREST STATEMENT

The authors declare no conflicts of interest.

## ETHICS STATEMENT

Ethical approval for the study (No. NRC24R/114/02) was granted by the Institutional Review Board (IRB) of King Abdullah International Medical Research Center (KAIMRC). Written informed consent was obtained from all patients prior to surgery, allowing the use of their data for research purposes. The consent included permission to publish anonymized data in academic settings.

## Data Availability

The data that support the findings of this study are available from the corresponding author upon reasonable request.
